# Proteomic Screening and Verification of Biomarkers in Different Stages of Mycosis Fungoides: A pilot Study

**DOI:** 10.3389/fcell.2021.747017

**Published:** 2021-12-13

**Authors:** Lu Gan, Haoze Shi, Ying Zhang, Jianfang Sun, Hao Chen

**Affiliations:** Institute of Dermatology, Chinese Academy of Medical Sciences and Peking Union Medical College, Nanjing, China

**Keywords:** mycosis fungoides, label-free quantitation, parallel reaction monitoring, pathogenesis, treatment

## Abstract

Mycosis fungoides (MF) is the most common cutaneous T-cell lymphoma; in advanced stages, it can involve multiple organs and has a poor prognosis. Early detection of the disease is still urgent, but there is no optimal therapy for advanced MF. In the present study, quantitative proteomic analyses (label-free quantitation, LFQ) were applied to tissue samples of different stages of MF and tissue samples from controls (eczema patients and healthy donors) to conduct preliminary molecular analysis to clarify the pathogenesis of the disease. Differential protein expression analysis demonstrated that 113 and 305 proteins were associated with the early and advanced stages of MF, respectively. Gene ontology (GO) enrichment analysis was conducted to determine the potential functions of the proteins, which could be classified into three categories: biological process, cellular component, and molecular function. The results revealed that a series of biological processes, including “initiation of DNA replication” and “nucleosome assembly,” were involved in the disease. Moreover, cellular components, including the “desmosome” and “integrin complex,” may affect the invasion and metastasis of MF via molecular functions, including “integrin binding” and “cadherin binding”. Kyoto Encyclopedia of Genes and Genomes (KEGG) pathway enrichment analysis demonstrated that “focal adhesion DNA replication,” “Toll-like receptor signalling pathway” and other pathways were also involved. A parallel reaction monitoring (PRM) assay was applied to validate the identified differentially expressed proteins. In conclusion, the above proteomic findings may have great diagnostic and prognostic value in diverse malignancies, especially MF. Nevertheless, further studies are still needed to explore the precise mechanisms of MF.

## Introduction

Mycosis fungoides (MF) is an extranodal, indolent non-Hodgkin lymphoma of T cell origin and is the most common subtype of cutaneous T- cell lymphoma (CTCL). According to the tumour-node-metastasis + blood (TNMB) staging system of lymphoma, MF is divided into early stage (stage ⅠA-ⅡA) and advanced stage (stage ⅡB-Ⅳ) subgroups. The increasing prevalence of MF over time has become a new challenge for the global health care system (Ghazawi et al., 2017; Keto et al., 2021). The early stage of MF can simulate a variety of inflammatory diseases, which makes accurate diagnosis relatively difficult (Olsen et al., 2007), while the advanced stage may involve a variety of organs, such as the lymph nodes, peripheral blood or viscera, leading to a poor prognosis (Agar et al., 2010; Trautinger et al., 2017). Moreover, the existing remedies based on multidrug chemotherapy also have poor long-term effects (Jawed et al., 2014). Hence, reliable biomarkers for early identification and rapid and effective treatments for patients with advanced-stage disease are still urgently needed.

In this study, label-free quantitation (LFQ) analyses were used to investigate the tissue proteomes of patients in different stages to find new biomarkers for early diagnosis and key molecules involved in the pathogenesis and progression of the disease. After preliminarily screening the differentially expressed proteins, parallel reaction monitoring (PRM) was applied to confirm the results.

## Materials and Methods

### Tissue Collection

Tissue specimens were collected from patients with MF or eczema and from healthy donors from August 2017 to January 2019 at the Institute of Dermatology, Chinese Academy of Medical Sciences and Peking Union Medical College (Nanjing, China). In this study, to investigate the key pathogenic factors of MF occurrence and progression from the early to advanced stage, we selected 3 healthy donors (group A) and 3 eczema patients (group B) as the control group and 3 patients with early-stage MF (group C) and 3 patients with advanced-stage MF (group D) as the main research objects. Clinical characteristics such as age, sex, clinical stage at the time of diagnosis, disease progression, and treatment were registered for each patient (Supplementary Table S1). The study was approved by the Ethics Committee of the Institute of Dermatology, Chinese Academy of Medical Sciences and Peking Union Medical College (approval no. 2013-LC/KY-033). All participating patients gave informed consent.

### Sample Preparation

The samples (6 from torso and 6 from limbs, each approximately 0.5 cm in diameter) were frozen in liquid nitrogen and ground with a mortar and pestle. Five volumes of TCA/acetone (1:9) were added to the powder and mixed by vortexing. The mixture was placed at −20°C for 4 h and centrifuged at 6,000 g for 40 min at 4°C. The supernatant was discarded. Precooled acetone was added and washed three times. The precipitation was air dried. Thirty volumes of SDT buffer were added to 20–30 mg powder, mixed and boiled for 5 min. The lysate was sonicated and then boiled for 15 min. After centrifugation at 14,000 *g* for 40 min, the supernatant was filtered with 0.22 µm filters. The filtrate was quantified with the BCA Protein Assay Kit (P0012, Beyotime). The sample was stored at −20°C.

### SDS-PAGE Separation

20 mg of protein from each sample was mixed with 6× loading buffer and boiled for 5 min. The proteins were separated on a 12.5% SDS-PAGE gel. The protein bands were visualized by Coomassie Blue R-250 staining.

### Filter-Aided Sample Preparation

Two hundred micrograms of protein for each sample was reduced with 50 mM DTT for 30 min at 56°C. The detergent, DTT and other low-molecular-weight components were removed using UA buffer (8 M urea, 150 mM Tris-HCl pH 8.5) by repeated ultrafiltration (Sartorius, 30 kD). Then, 100 μL iodoacetamide (100 mM IAA in UA buffer) was added to block reduced cysteine residues, and the samples were incubated for 30 min in darkness. The filters were washed with 100 μL UA buffer three times and then 100 μL 25 mM NH4HCO3 buffer twice. Finally, the protein suspensions were digested with 4 μg trypsin (Promega) in 40 μL 25 mM NH4HCO3 buffer overnight at 37°C, and the resulting peptides were collected as the filtrate.

### Mass Spectrometry Analysis

The peptides of each sample were desalted on C18 cartridges, concentrated by vacuum centrifugation and reconstituted in 40 µL of 0.1% (v/v) formic acid. The peptide content was estimated according to the UV light spectral density at 280 nm using an extinction coefficient of 1.1 of 0.1% (g/L) solution that was calculated on the basis of the frequency of tryptophan and tyrosine in vertebrate proteins. LC-MS/MS analysis was performed on a Q Exactive Plus mass spectrometer (Thermo Fisher Scientific) that was coupled to Easy nLC (Thermo Fisher Scientific). Two micrograms of peptide was loaded onto a C18 reversed-phase analytical column (Thermo Fisher Scientific, Acclaim PepMap RSLC 50 μm × 15 cm, nanoViper, P/N164943) in buffer A (0.1% formic acid) and separated with a linear gradient of buffer B (80% acetonitrile and 0.1% formic acid) at a flow rate of 300 nL/min. The linear gradient was as follows: 5% buffer B for 5 min, 5–28% buffer B for 90 min, 28–38% buffer B for 15 min, 38–100% buffer B for 5 min, and holding in 100% buffer B for 5 min. MS data were acquired using a data-dependent top 10 method, which dynamically selects the most abundant precursor ions from the survey scan (350–1800 m/z) for higher energy collisional dissociation (HCD) fragmentation. MS1 scans were acquired at a resolution of 70,000 at m/z 200 with an AGC target of 3e6 and a max IT of 50 ms. MS2 scans were acquired at a resolution of 17,500 at m/z 200 with an AGC target of 2e5 and a max IT of 45 ms, and isolation width was 2 m/z. Only ions with a charge state between 2-6 and a minimum intensity of 2e3 were selected for fragmentation. Dynamic exclusion for selected ions was 30 s. Normalized collision energy was 27 eV.

### Data Analysis

MaxQuant software (version 1.5.5.1) was used for database search, and LFQ (Label-Free Quantitation) algorithms (Cox et al., 2014) were used for quantitative analysis. Relevant parameters and instructions were as follows (Table 1). Proteins with fold change>1.2 and *p* value (Student’s *t* test) < 0.05 were considered to be differentially expressed proteins.

**TABLE 1:** Relevant parameters and instructions for LFQ.

Item	Value
➢Enzyme	• Trypsin
➢Max missed cleavages	• 2
➢Main search	• 4.5 ppm
➢First search	• 20 ppm
➢MS/MS Tolerance	• 20 ppm
➢Fixed modifications	• Carbamidomethyl (C)
➢Variable modifications	• Oxidation (M), Acetyl (Protein N-term)
➢Database	• Uniprot_HomoSapiens_20,386_20180905
➢Database pattern	• Target-Reverse
➢Include contaminants	• True
➢Peptide FDR	• ≤0.01
➢Protein FDR	• ≤0.01

### Gene Ontology Annotation

First, all protein sequences were aligned to the Linux database downloaded from NCBI (ncbi-blast-2.3.0+), and only the sequences in the top 10 with E-value ≤ 1*e*-3 were retained. Second, the GO term (database version: go_201,504. obo) of the sequence with the top bit score by Blast2GO was selected (http://www.geneontology.org). Then, the annotation of GO terms to proteins was completed with Blast2GO Command Line. After preliminary annotation, InterProScan (Quevillon et al., 2005) was used to search the EBI database by motif and then add the functional information of motifs to proteins to improve the annotation process. Then, further improvements regarding annotation and connection between GO terms were made with ANNEX. Fisher’s exact test was used to assess enriched GO terms by comparing the number of differentially expressed proteins and total proteins correlated with the GO terms.

### Kyoto Encyclopedia of Genes and Genomes Pathway Annotation

Pathway analysis was performed using the KEGG database and KEGG Orthology And Links Annotation (KOALA) software (Kanehisa et al., 2016). Fisher’s exact test was used to identify the significantly enriched pathways by comparing the number of differentially expressed proteins and total proteins correlated with the pathways.

### Clustering

The quantitative information for target protein collection was first normalized. Second, Matplotlib software was used to classify the expression levels of samples and proteins (distance algorithm: Euclidean, linkage method: average linkage) and generate the hierarchical clustering heat map.

### Trend Analysis

Short Time-Series Expression Miner (STEM) (Ernst and Bar-Joseph 2006) was used to carry out significant analysis on the up- or downregulated proteins to obtain trend models with significant changes.

#### Parallel Reaction Monitoring

PRM-MS analysis was conducted following the manufacturer’s (Peterson et al., 2012) method, aiming to verify the discovery results. The enzymatic hydrolysate was separated by a nano-UPLC liquid phase system (EASY-nLC1200) and detected using a Q-Exactive Plus mass spectrometer (Thermo Fisher) for MS. Samples were directly loaded onto a 100 μm ID × 15 cm reversed-phase chromatographic column (Reprosil-Pur 120 C18) and separated. Mobile phase A was a 0.1% formic acid aqueous solution, and liquid B was a 0.1% formic acid acetonitrile aqueous solution (acetonitrile was 80%). The column was balanced with 100% liquid A. The flow rate was 300 nL/min, and the gradient time was 120 min. The parameters for mobile phase B were as follows: 6–28% for 92 min, 28–40% for 20 min, 40–100% for 2 min, 100% for 2 min, 2% for 2 min. The LC-MS/MS parameters were as follows: analysis time of 120 min/sample, positive ion detection mode. After nano-UPLC separation, PRM data were collected by MS and imported into Skyline for transition extraction (method match tolerance: 0.055 m/z). The above experiments were performed at Kangcheng Biotech (Shanghai, China). The thresholds for identification of the differentially expressed peptides were set at *p* < 0.05 and fold-change >2.

## Results

### Differentially Expressed Protein Profiles Detected by Label-Free Quantitation Analysis

**FIGURE 1:**
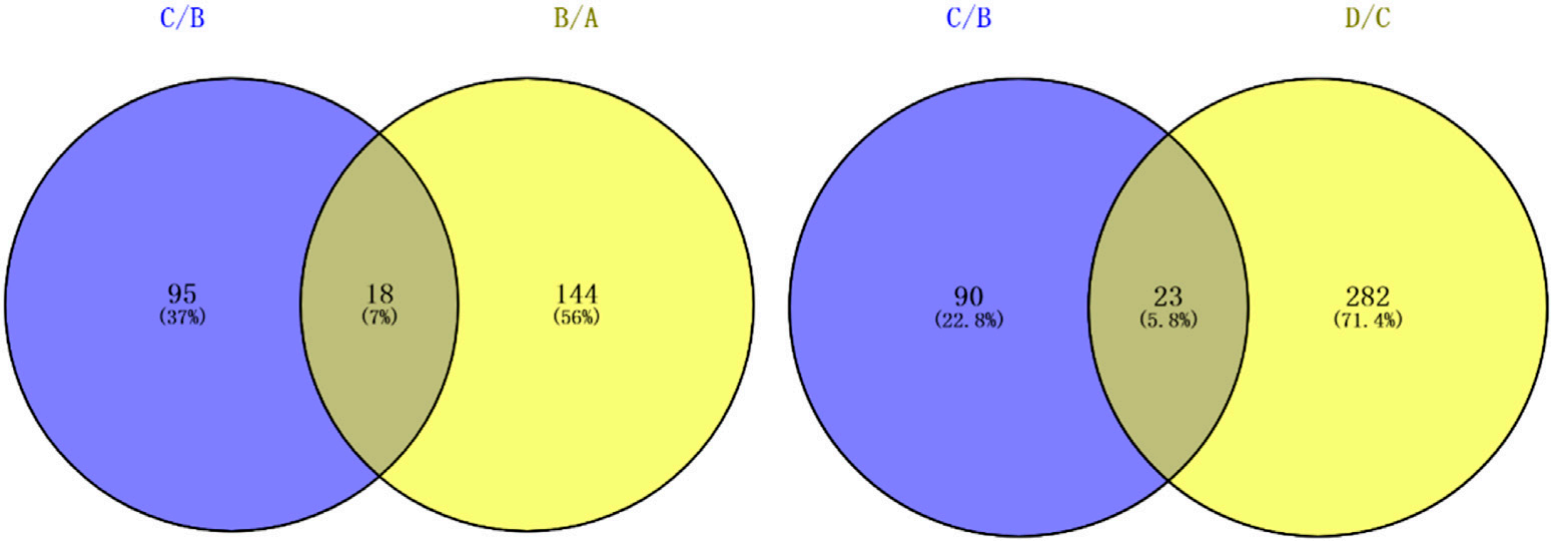
Venn diagrams show the related pathogenic proteins overlapping among groups.

**FIGURE 2:**
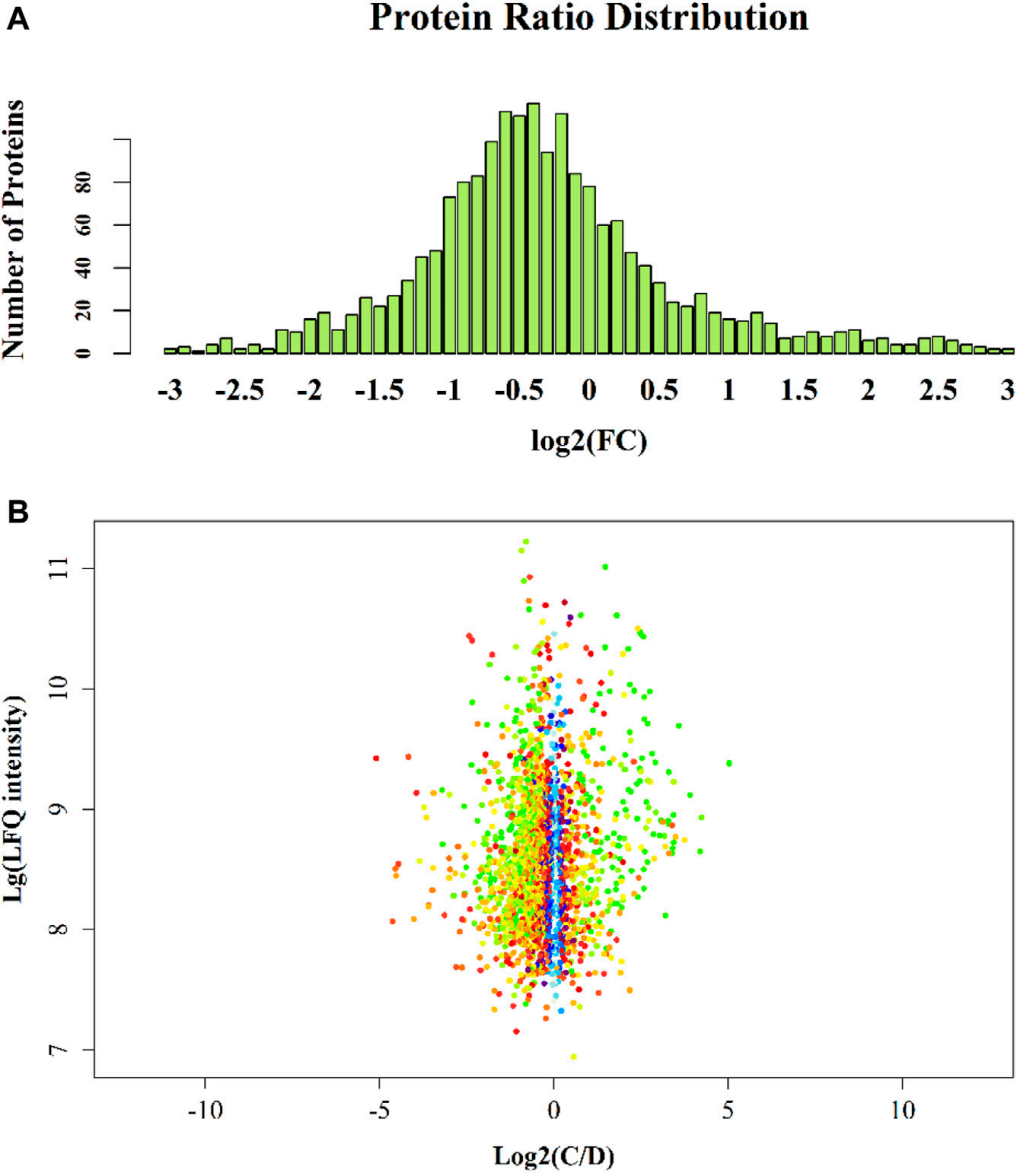
Group C vs D protein abundance diagram. Note: The abscissa is the fold change (log base 2-transformed); the ordinate is the number of identified proteins. Figure 2B Group C vs D scatter plot. Note: The variations in the protein expression profiles between group C and D tissue samples were assessed, and scatter plots were constructed to demonstrate the association between the fold changes and LFQ intensities of the differentially expressed proteins. Each data point is marked with a different colour according to the *p* value obtained by the *t* test algorithm: blue represents *p* value >0.05; red represents 0.01 < *p* value <0.05; yellow represents 0.001 < *p* value <0.01; green represents *p* value <0.001. The abscissa is the fold change (log base 2-transformed); the ordinate is the sum of LFQ intensity (log base 10-transformed).

There were significantly differentially expressed proteins in the A, B, C, and D groups (Table 2). In total, 113 significantly differentially expressed proteins (80 upregulated and 33 downregulated) were identified from the C vs B comparison; 305 significantly differentially expressed proteins (196 upregulated and 109 downregulated) were identified from the D vs C comparison. As shown in Figure 1, excluding the overlapping 18 proteins (7%), 95 differentially expressed proteins (37%) between groups C and B, represented the occurrence of the disease. The 23 overlapping proteins (5.8%) between groups C and B and groups D and C groups represented the development of the whole disease process (Table 3). The variations in the protein expression profiles between groups C and D were assessed with a protein abundance diagram (Figure 2A), and scatter plots were generated to demonstrate the association between the fold changes and the statistical significance of the differentially expressed proteins (Figure 2B).

**TABLE 2:** Quantitative protein results by LFQ analysis.

Comparisons	Up-	Down-	All-
B/A	93	69	162
C/A	130	53	183
D/A	281	88	369
C/B	80	33	113
D/B	281	133	414
D/C	196	109	305

Note: Comparison: groups compared; up: upregulated proteins; down: downregulated proteins; all: all differentially expressed proteins.

**TABLE 3:** Twenty-three significantly overlapping proteins were identified between the C/B and D/C groups by LFQ analysis.

Main protein ID	Gene name	Fc (C/B)	*p* value (C/B)	Fc (D/C)	*p* value (D/C)
Q96Q06	PLIN4	9.6259	0.01693	0.0941	0.0155
P30838	ALDH3A1	3.3178	0.0383	0.0546	0.0360
Q9BXN1	ASPN	2.5884	0.0396	0.3299	0.0032
P07305	H1F0	2.5297	0.0053	0.2873	0.0048
O15533	TAPBP	2.5275	0.0129	1.6607	0.0437
P43121	MCAM	2.0192	0.0135	0.4952	0.0135
Q9UIJ7	AK3	1.9716	0.0423	0.5223	0.0228
Q96AC1	FERMT2	1.8746	0.0106	0.4781	0.0131
P55268	LAMB2	1.8675	0.0390	0.3909	0.0278
Q13642	FHL1	1.7952	0.0065	0.2600	0.0038
Q9HBL0	TNS1	1.7611	0.0271	0.2845	0.0039
Q9NYU2	UGGT1	1.7197	0.0280	1.4311	0.0314
P62993	GRB2	1.5361	0.0076	3.5980	0.0191
Q12874	SF3A3	1.5010	0.0081	2.7120	0.0377
Q07666	KHDRBS1	1.4771	0.0351	1.5029	0.0204
P05455	SSB	1.4291	0.0456	1.5436	0.0020
O00159	MYO1C	0.0033	0.0155	0.5050	0.0033
Q08945	SSRP1	1.3621	0.0028	3.1920	0.0346
P61978	HNRNPK	1.3050	0.0455	1.3363	0.0429
O75369	FLNB	0.7404	0.0336	0.5533	0.0158
P41250	GARS	0.6821	0.0359	1.6045	0.0094
Q13835	PKP1	0.6248	0.6248	0.6248	0.0009
Q9Y6G9	DYNC1LI1	0.5905	0.0051	1.4122	0.0283

Note: The table shows the main protein IDs, gene names; LFQ, intensities, fold changes, and *p* values.

### Gene Ontology and Kyoto Encyclopedia of Genes and Genomes Pathway Enrichment Analysis

**FIGURE 3:**
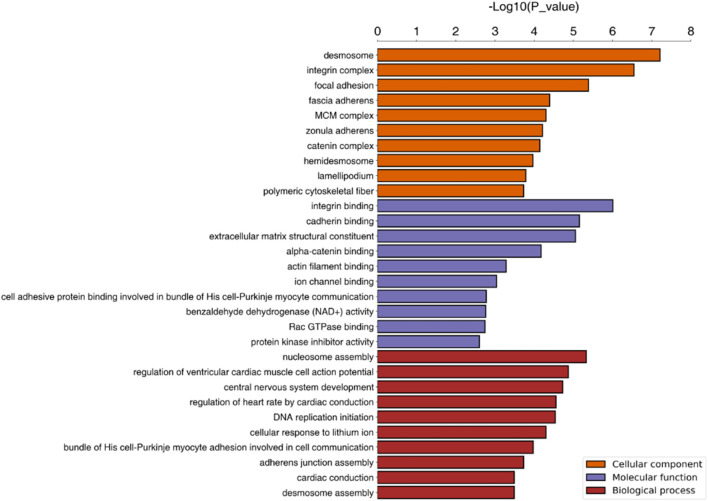
GO enrichment analysis. Note. The top 10 terms associated with differentially expressed proteins are presented. BP, biological process; CC, cellular component; GO, gene ontology.

**FIGURE 4:**
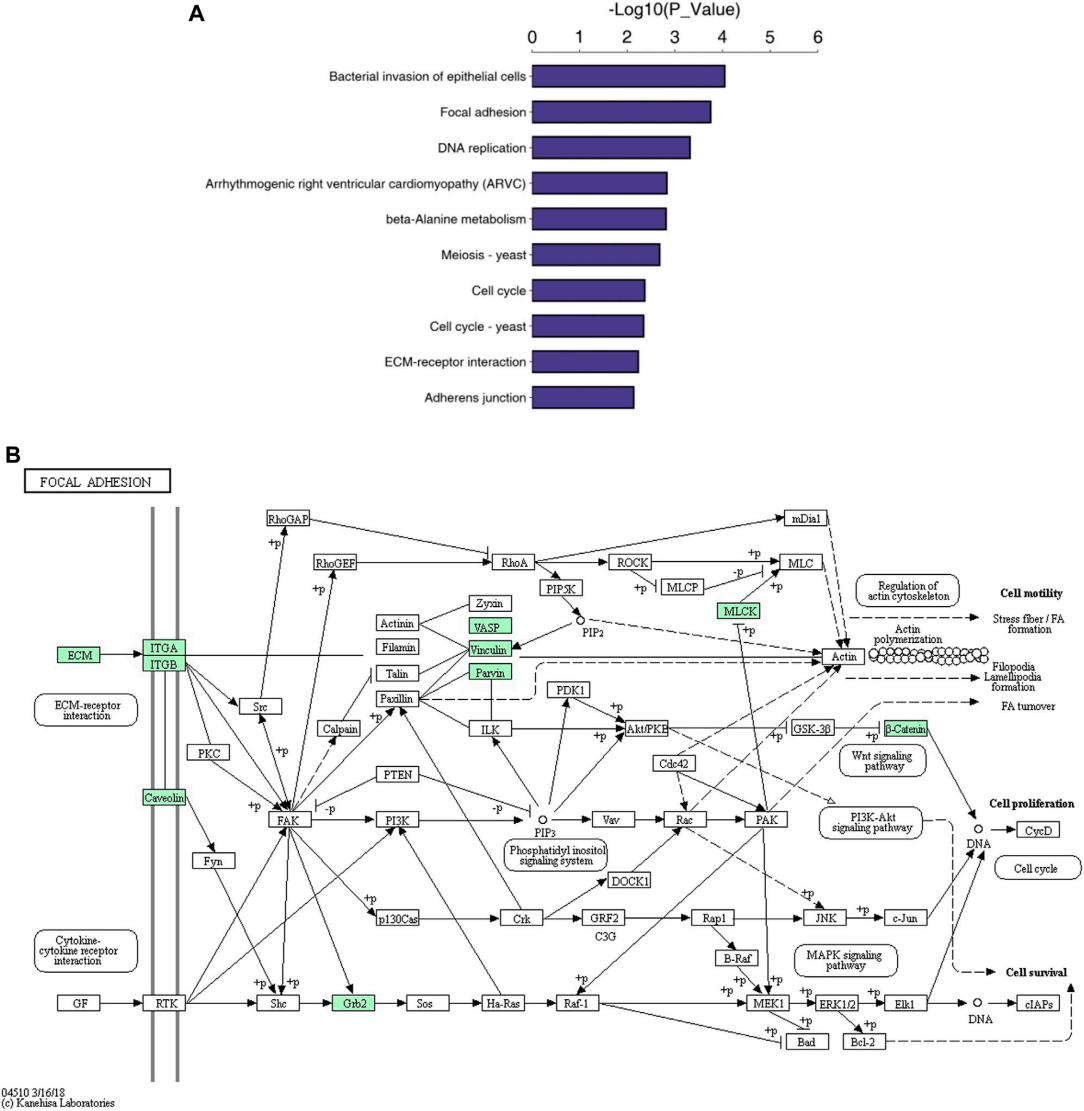
KEGG pathway analysis. Note. The top 10 pathways of differentially expressed proteins **(A)** and the focal adhesion pathway **(B)**.

The cellular component (CC), biological process (BP) and molecular function GO categories were analysed to determine the gene and gene product enrichment. The results of GO enrichment analysis between groups C and D are presented in Figure 3; the main enriched BPs were involved in cellular and biological processes, including “DNA replication initiation,” “nucleosome assembly” and “cellular response to external stimulus”. With regard to CCs, most of these proteins were associated with “desmosomes,” the “integrin complex” and “focal adhesion”. Terms associated with binding activity, especially “integrin binding,” “cadherin binding,” “alpha-catenin binding” and “extracellular matrix (ECM) structural constituent,” were enriched in the molecular function category. The results of KEGG pathway enrichment analysis are presented in Figure 4A and indicated the involvement of differentially expressed proteins in “bacterial invasion of epithelial cells,” “focal adhesion DNA replication,” “Toll-like receptor signalling pathway” and “beta-alanine metabolism.” Among them, a number of metabolic pathways were commonly found, and the focal adhesion process was particularly enriched (Figure 4B).

### Clustering

**FIGURE 5:**
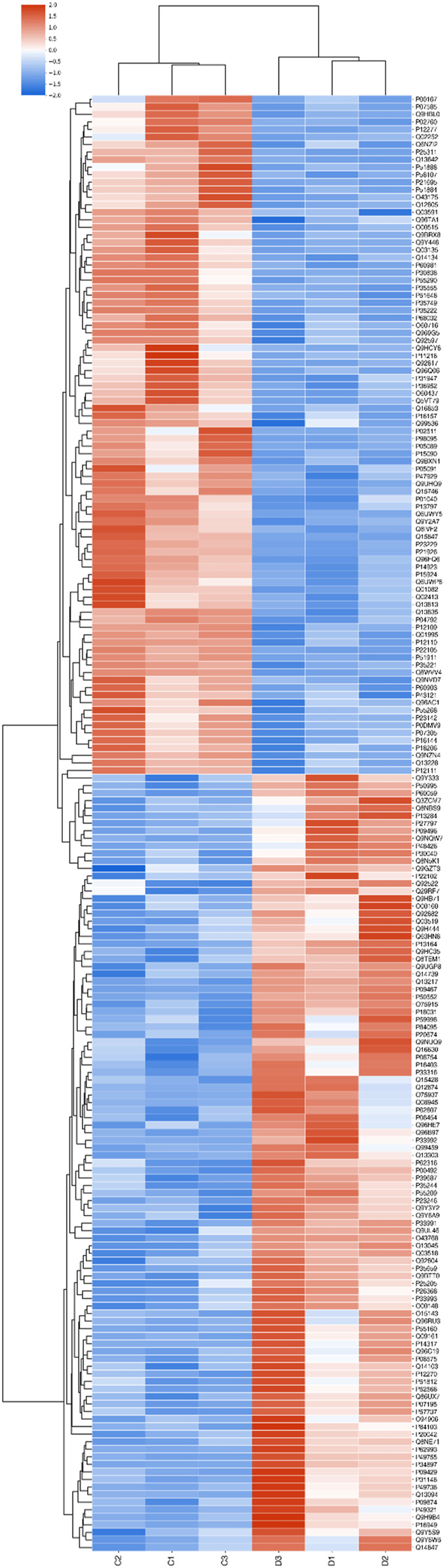
Hierarchical clustering. Note. Proteins differentially expressed in groups C and D were subjected to hierarchical clustering. The samples were divided into two groups according to the expression levels of these proteins; red indicates high relative expression, and blue indicates low relative expression. The brightness is positively correlated with the fold change.

The expression patterns of the differentially expressed proteins were demonstrated in hierarchical clustering (Figure 5).

### Trend Analysis

**FIGURE 6:**
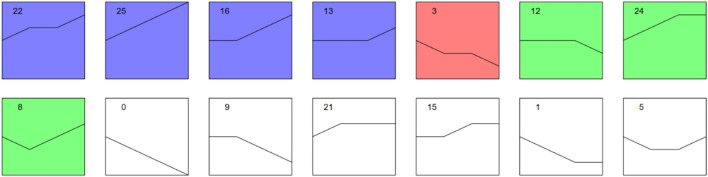
Trend models with significance of protein expressions. Note: Each block diagram in the figure represents a trend profile. The number in the upper left corner is the number of profiles. Different groups were labelled by STEM according to the trend and significance level of each profile. Those with the same colour showed similar significant trends, while those without colour showed nonsignificant trends. STEM, Short Time-Series Expression Miner.

Different trend models with significant changes in up- or downregulated proteins were carried out as follows (Figure 6, Supplementary Figures S1–2, Supplementary Tables S1A–S2A).

### Parallel Reaction Monitoring

**FIGURE 7:**
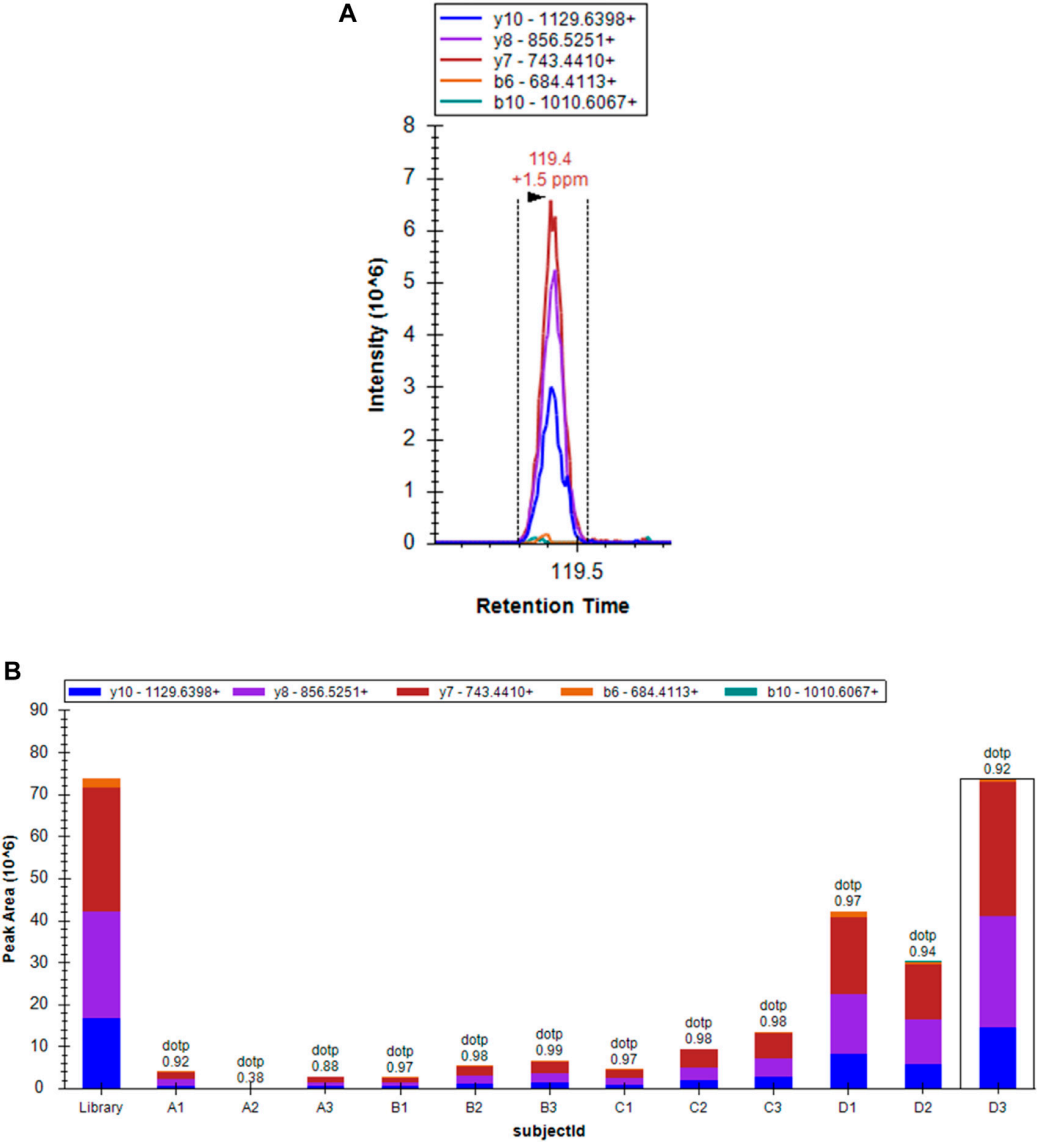
Spectral peak figure shows the expression of MCM3 in the sample D3 Figure 7B Histogram showing the expression of MCM3 in different groups.

To confirm the previous results and detect the function of proteins expressed in both the early and the advanced stage of MF, 50 selected proteins (Supplementary Table S3) were validated by PRM; these proteins included those with significant changes and those enriched in some specific pathways and components based on the results of bioinformatics analysis. As shown in Table 4 and Figure 7, the concentration of the selected proteins was significantly higher (*p* < 0.05) in group D than in group C, confirming that there was a clear overproduction of related proteins with the progression of the disease. The above findings serve as reliable confirmation of the protein changes determined by LFQ.

**TABLE 4:** Detailed lists of significantly expressed proteins.

Protein Name	Fold change (D/C)	p.value (D/C)
SERPINB5	2.422824979	0.010797906
SSRP1	2.267709861	0.044248565
ITGB4	0.375482894	0.031100313
COL6A1	0.170598304	0.036254626
PLIN4	0.170637917	0.00050838
MCM3	3.345329674	0.03005326
TNXB	0.189852295	0.030431991
MCM5	6.908794219	0.028524297
UGGT1	2.645307058	0.038085875
KHDRBS1	0.351991202	0.017846017
MCM2	6.195499826	0.034073626
STMN1	4.636626485	0.020608702
H1F0	0.417768823	0.046817625
ITGA6	0.472315349	0.024424589
ALDH3A1	0.132337909	0.00699105
CD44	1.85356264	0.04913183

## Discussion

MF is a mature T cell non-Hodgkin lymphoma with presentation in the skin but with potential involvement of the nodes, blood, and viscera in the advanced stage, leading to a poor prognosis. To further explain the mechanism and malignant biological behaviours of MF, quantitative proteomic analyses and confirmatory tests were performed on different stages of MF. Moreover, GO and KEGG pathway enrichment analyses were conducted to highlight the significantly differentially expressed proteins and determine their potential functions.

As mentioned above, differential expression analysis demonstrated that 113 and 305 proteins were associated with early and advanced MF, respectively. In addition, we speculated that the remaining 95 proteins that differed between groups C and B may indicate the occurrence of the disease, while the overlapping 23 proteins may help predict the progression and prognosis of MF. GO enrichment analysis was used to identify the functions of the proteins and their roles in the progression of the disease. Relevant proteins that were up- or downregulated and their functions in multiple malignancies are summarized below (Table 5).

**TABLE 5:** Relevant proteins and their functions in GO enrichment analysis.

GO term	Category	*p* value	Gene names	Mechanisms	Malignancies	References
Integrin complex	CC	<0.0001	TNXB, MYH11, ITGA6, ITGB4, DSP, etc.	These factors mediate physical interactions with the ECM and regulate the proliferation, migration, and invasion of tumour cells by inhibiting the phosphorylation of series of downstream signalling targets, increasing the expression of MMP-2 and MMP-9, and reducing the expression of caspase-3	Ovarian cancer, cervical cancer, breast cancer, lung cancer	Desgrosellier and Cheresh 2010; Choi et al., 2012; Yan et al., 2019
Focal adhesion	CC	<0.0001	EVPL, MYH11, CRYAB, CNN1, ANXA8LA, EPPK1, etc.	These factors increase to the activity of ERK1/2, whose secretion is promoted by MMP-9, or upregulate PI3K and Akt and were positively correlated with cell invasiveness	Breast cancer, lung cancer, prostate cancer, ovarian cancer, etc.	Hwang et al., 2008; Ni et al., 2008, Ansell and Lin 2020; Eke and Cordes 2015; Zhang et al. (2019)
MCM complex	CC	<0.0001	MCM2, MCM3, MCM4, MCM5	These factors play different roles in cellular processes such as: chromatin remodelling, DNA repair, DNA transcription, RNA processing, and cell cycle regulation	Adrenocortical cancer, CTCL, oral cancer	Aporowicz et al., 2019; Hyrien 2016; Jankowska-Konsur et al., 2015; Niranjan et al. (2018)
Integrin binding	MF	<0.0001	FBLN1, FBLN2, FBN1, ITGA6, DSP, TNXM, etc.	These factors belong to a family of extracellular glycoproteins that modulate cell morphology, cellular interaction with the ECM and cell migration	Colorectal cancer, urothelial cancer, lung cancer, ovarian cancer, etc.	Desgrosellier and Cheresh 2010, Choi et al., 2012, Watany et al., 2018
Cadherin binding	MF	<0.0001	JUP, CTNNA1, CTNNB1, ANK1, CDH13, NDRG1, etc.	The cytoplasmic tail of E-cadherin is associated with various catenins (α, β, and p120) that link to the cytoskeleton and mediate downstream signalling including the Hippo, Wnt, TGFβ, NF-κB, and other growth factor signalling pathways	Bladder cancer, gastric cancer, breast cancer	Mendonsa et al., 2018; Chi et al., 2020; Clark et al., 2020; Mendonsa et al. (2018)
ECM structural constituent	MF	<0.0001	FBLN1, FBLN2, PRELP, COL6A1, COL6A2, LAMB2, etc.	These factors stimulate cell motility and support distant colonization by regulating the expression of oncogenic transcription factors, tumour suppressor p53, and SMAD family member 4	Prostate cancer, renal cancer, cervical cancer, osteosarcoma	Edoo et al., 2019; Hou et al., 2016; Birnbaum et al., 2011
Nucleosome assembly	BP	<0.0001	H1F0, H1FX, HMGB1, HIST1H2BC, H1ST1H1C, NAP1L1, etc.	These factors enhance proliferation while inhibiting the apoptosis of cancer cells through the upregulation of MMP-9 activity	Gastric cancer, bladder cancer	Rochman et al., 2010; Liu et al., 2019; Wahafu et al., 2011
DNA replication initiation	BP	<0.0001	MCM2, MCM3, MCM4, MCM5, MCM7	MCM-3 is phosphorylated by cyclin B/CDK1 and plays a regulatory role in MCM2–7 complex. MCM-5, in cooperation with cyclin E, associates with the centrosome and regulates its duplication. MCM-7 interacts with many molecules involved in cell cycle regulation, including pRb, Mat-1 and FLH.	Adrenocortical cancer, CTCL, oral cancer	Lan and Wang 2020; Aporowicz et al., 2019; Hyrien 2016; Niranjan et al., 2018; Jankowska-Konsur et al., 2015
Adherens junction assembly	BP	0.0002	JUP, VCL, CTNNB1	As cell-cell junction proteins, they are involved in adhesion junction and desmosome composition, the loss of which results in increased *p*-AKT levels and AKT/GSK3β/β-catenin signalling activity	Colon cancer, gastric cancer	Peterson et al., 2012; Lan and Wang 2020; Hristov et al., 2019

The enriched CC terms were associated with CCs and molecules connected with the extracellular region, especially the “integrin complex,” “focal adhesion,” and the “MCM complex.” As in previous studies, integrins and integrin-linked kinase (ILK) were essential for invasion because they mediated physical interactions with the ECM and regulated oncogenic signalling pathways (Desgrosellier and Cheresh 2010; Choi et al., 2012). Targeting integrin β4 (ITGB4) combined with ILK could increase the latent tumorigenic potential and decrease the invasive potential in cancer. In CTCL, activation of T-cell integrins facilitates T-cell adhesion to skin endothelial cells and subsequent binding to ECM proteins, which regulates a diverse array of cellular functions and modulates the tumour microenvironment (Hwang et al., 2008; Ni et al., 2008; Ansell and Lin, 2020). During the process, focal adhesion kinase (FAK), which localizes at contact points of cells with the ECM, also plays a critical role in cell survival, motility and metastasis and is upregulated in numerous types of human tumours (Eke and Cordes, 2015). In addition, the CC terms of the MCM complex were particularly prominent. Normally, the cellular levels of MCM proteins depend on the cell status (their levels are higher in proliferating cells and much lower in quiescent, differentiated or senescent cells). This makes MCM proteins useful markers of cell proliferation, and therefore, evaluation of MCM levels could help detect various precancerous states and preinvasive and invasive neoplasms. Numerous studies have pointed out that MCM is more specific and sensitive than conventional proliferative markers such as Ki-67 and PCNA.

In terms of molecular function, “protein binding” and “enzyme binding” terms including “cadherin binding,” “ECM structural constituent,” and “integrin binding” were enriched among the differentially expressed proteins. These proteins and their downstream signalling pathways mainly influence the regulation of contact inhibition. Among them, E-cadherin is a key component of adherens junctions, the loss of which results in increased cell motility and cancer progression (Mendonsa et al., 2018). Other significantly differentially expressed proteins included collagen type VI alpha 1 (COL6A1), which is localized in the ECM and involved in cell adhesion and collagen remodelling (Cescon et al., 2015; Lamande and Bateman 2018). COL6A1 may play an important role in the metastatic process and could be considered a predictor of poor outcomes in several cancers (Edoo et al., 2019; Hou et al., 2016). Notably, some studies have revealed that the transcription factor c-Jun-bound p300 increases the enrichment of H3K27ac at the promoter region of COL6A1, thus resulting in the upregulation of COL6A1 in osteosarcoma (OS). Furthermore, COL6A1 promotes OS metastasis by suppressing STAT1 by promoting its ubiquitination proteasome degradation (Zhang et al., 2021). In our study, the trend in STAT1 expression was consistent with the above findings. Therefore, we speculated that COL6A1, as a link between the H3K27 acetylation process and the JAK-STAT pathway, might serve as a new therapeutic target for MF.

We found that most of the BP terms were associated with “DNA replication initiation” and “nucleosome assembly.” Among these proteins, MCM family proteins are vital for the process of DNA replication and are often called replication-licensing proteins. Additionally, the regulation of DNA replication is closely connected with the histone acetylation process. As was found in this study, with the progression of tumours, the difference in HDAC1 gradually became significant. To date, a variety of histone deacetylase inhibitors (HDACis) have been used clinically in the treatment of T-cell lymphoma (Hristov et al., 2019). Therefore, further studies focused on the oncogenic roles of these processes and the potential use of HDACis for the treatment of MF are needed. In addition, the process of “nucleosome assembly,” which is affected by multiple proteins—mainly the high mobility group nucleosome-binding (HMGN) proteins, modulates the cellular epigenetic profile, changes gene expression and impacts biological processes such as development and the cellular response to environmental and hormonal signals (Postnikov and Bustin 2010). Thus, the HMGN family and its regulatory pathways may play a positive role in cell proliferation and invasion in lymphoma.

KEGG pathway enrichment analysis revealed that pathways such as ‘focal adhesion’, “ECM-receptor interaction,” “adherens junctions,” “DNA replication,” the “cell cycle” and the “bacterial invasion of epithelial cells” were closely associated with the differentially expressed proteins. The migration, invasion and metastasis of tumour cells lead to tumorigenesis. Multifunctional focal adhesion complexes facilitating cell-ECM contact and the connection between the ECM and actin cytoskeleton play mechanistically key roles, as they structurally and functionally control cell morphology and cytoplasmic signalling. During this process, focal adhesion kinase plays an important role in Erb-2/Erb3-induced oncogenic transformation and invasion and can block the p53 transcriptional activity of p21, BAX and Mdm-2 (Benlimame et al., 2005; Golubovskaya and Cance 2010). Moreover, as discussed above, MCM family proteins interact with many molecules involved in DNA replication and cell cycle regulation (Hyrien 2016; Aporowicz et al., 2019). We compared our results with those of previous studies (Andrews et al., 2019), and we found that genes with significant changes were related to the apoptosis, cell cycle, cytokine/chemokine signalling, especially the ECM and cell adhesion/migration pathways, consistent with our results; these genes included MCM2, MCM4, CDK1, CDK6 (cell cycle), CDK1, CDK6, PERP, RRM2, SERPINB5 (p53 signalling pathway), ITGB4, ITGA6, LAMA5 (ECM-receptor interaction), and CFH, CAMP, C1QC (bacterial invasion of epithelial cells). These genes may play a role in the variable manifestations of MF in the skin, lymph nodes, and peripheral blood.

The results verified by PRM were consistent with those of the quantitative proteomic analysis, which proved the reliability of the above analysis. We also identified a series of meaningful proteins that have been previously found to be associated with MF. Networks including MAPK, PI3K/Akt, JAK/STAT, NF-κB, TCR, and TLR downstream signalling were activated in malignant T-cells, facilitating ECM remodelling and tumour progression (Bastidas et al., 2018; Seto et al., 2018; Kamijo et al., 2018; Perez et al., 2020; Rendon-Serna et al., 2021). The screening of ZAP70 also drew our attention, as it was recently found to be a regulatory point of non-Hodgkin lymphoma that acts via the Ras-Raf-dependent pathway and to induce cell apoptosis (Bharti et al., 2016). In particular, although STAT1, STAT2, STAT3, STAT5, and STAT6 were screened in our study and were already reported to be involved in several MF studies, they did not show significant expression trends among the compared groups but increased significantly in group B, reflecting their involvement in inflammatory diseases (Bastidas et al., 2018; Garcia-Colmenero et al., 2020; Gaydosik et al., 2020; Rendon-Serna et al., 2021).

In this study, through intergroup comparison and trend analysis, we also revealed other meaningful results that may explain the mechanism and progression of MF from a new perspective. In some trend models, we found that the expression of several proteins was initially not significantly different according to our thresholds but gradually changed remarkably with the progression of the disease. In profile 22, DDX39A was apparently overexpressed after a plateau. As a new member of the DEAD box RNA helicases, it plays a role in RNA splicing/export and is involved in the pathogenesis of cancers, including bladder cancer (Sugiura et al., 2007; Kato et al., 2012). In addition, it was found to be positively related to a poor prognosis via the regulation of cell proliferation, G2/M cell cycle arrest, caspase-mediated cell apoptosis, and cell invasion and migration in melanoma (Xing et al., 2020). Haematopoietic cell-specific lyn substrate-1 (HCLS1), another protein in profile 22, is important for the activation of GTPases and integrins and mediates the downstream signalling of many receptors, including BCR, TCR, and CXCR4 (Castro-Ochoa et al., 2019). In profile 25, PARP1, as the main poly (ADP-ribose) polymerase molecule, could influence many processes, including genome maintenance, replication, transcription, and chromatin remodelling, which makes it a central regulatory hub in many cancer-relevant processes (Engbrecht and Mangerich, 2020). Proteomic analyses in aggressive MF demonstrated that PARP-1 was overexpressed in patients with early-stage MF and may serve not only as a biomarker at initial biopsy for a disease that may become aggressive but also as a new therapeutic target for advanced MF(Lemchak et al., 2018). In addition, heat-shock protein family A (Hsp70) member 1 like (HSAP1L), Hsp70 member 1A (HSPA1A), and ATP-dependent RNA helicase (DDX17) had higher expression in aggressive disease versus nonaggressive (Lemchak et al., 2018), similar to what we found in this study.

In conclusion, the present study was conducted to compare protein expression in different stages of MF, and to screen out and verify the differentially expressed proteins. The results revealed that genes and proteins implicated in DNA replication initiation, nucleosome assembly, and cell adhesion activity, together with the CCs and molecules that connect extracellular regions may be involved in the pathogenesis and metastasis of MF via the regulation of apoptosis, the cell cycle, and cell adhesion/migration pathways. Some special proteins showed obvious differences from early -stage to advanced-stage MF, indicating that they may be the key molecules related to the progression of the disease. Research on the pathogenesis and treatment of MF is still worth furtherstudy. We believe that proteomics is a powerful tool to identify potential biomarkers for diagnosis and that these molecules may be promising therapeutic targets for MF. Further studies are still needed to determine their application value.

### Limitations of Our Study

The present study only involved an LFQ analysis and subsequent GO analysis, KEGG analysis, clustering analysis, trend analysis, and an initial verification by PRM. The sample size was relatively small, and further research on the function of the target proteins could be performed in our future studies to substantiate these findings.

## Data Availability

The original contributions presented in the study are publicly available. This data can be found here: ProteomeXchange dataset PXD027714.
