# Supplementary material for: Proteomic Screening and Verification of Biomarkers in Different Stages of Mycosis Fungoides: A pilot Study
Source: Front Cell Dev Biol. 2021 Dec 13;9:747017. doi: 10.3389/fcell.2021.747017 (PMC8711087; doi:10.3389/fcell.2021.747017)
Supplement: Supplementary file 1 [file DataSheet1.docx]

**Supplementary**

**Supplementary Table S1. Characteristics of the patients with mycosis fungoides in the study**

| NO. | Sex | Age | Duration | LM^1^ | LCT^2^ | TNMB | Clinical stage | Biopsy | Treatment |
| --- | --- | --- | --- | --- | --- | --- | --- | --- | --- |
| 1 | M | 44 | 1Y | - | - | T1N0M0B0 | ⅠA | Upper limb | Topical medication^3^ |
| 2 | F | 61 | 10Y | - | - | T2N0M0B0 | ⅠB | Lower  limb | Topical medication^3^ |
| 3 | M | 40 | 2Y | - | - | T2N0M0B0 | ⅠB | Back | Topical medication^3^ |
| 4 | M | 56 | 5Y | - | - | T3N0M0B0 | ⅠIB | Abdomen | Methotrexate+ Interferon+ Topical medication^3^ |
| 5 | M | 34 | 3Y | - | - | T3N0M0B0 | ⅡB | Abdomen | Oral and topical steroids |
| 6 | F | 47 | 6Y | + | + | T3N1M0B0 | ⅠIB | Back | Acitretin+ Superficial X-ray therapy+ Topical medication^3^ |

Abbreviation:

LM^1^, Lymphatic metastasis

LCT^2^, Large cell transformation

Topical medication^3^, Topical steroids and nitrogen mustard


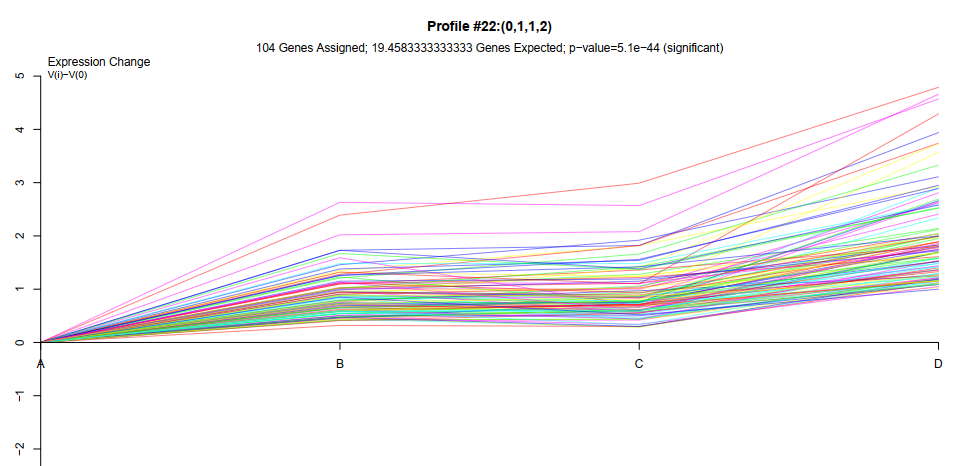


**Supplementary Figure S1. Trend model of protein expressions (Profile #22)**

Note: Each trend model has a graph corresponding to it. In the graph, each line represents a protein which visually reflect the expression changes in the trend model.

**Supplementary Table S1A. Detailed lists of proteins in the trend model (Profile #22)**

| **Proteins** | **Gene Name** | **SPOT** | **Profile** | **A** | **B** | **C** | **D** |
| --- | --- | --- | --- | --- | --- | --- | --- |
| O00148 | *DDX39A* | ID_677 | 22 | 0 | 2.39 | 2.99 | 4.79 |
| P14317 | *HCLS1* | ID_517 | 22 | 0 | 2.02 | 2.08 | 4.66 |
| P33992 | *MCM5* | ID_424 | 22 | 0 | 1.32 | 1.1 | 4.29 |
| P52566 | *ARHGDIB* | ID_349 | 22 | 0 | 1.28 | 1.82 | 3.74 |
| P31146 | *CORO1A* | ID_435 | 22 | 0 | 0.88 | 1.4 | 3.73 |
| Q08945 | *SSRP1* | ID_244 | 22 | 0 | 1.21 | 1.66 | 3.33 |
| Q9UL46 | *PSME2* | ID_35 | 22 | 0 | 1.46 | 1.92 | 3.11 |
| Q03519 | *TAP2* | ID_258 | 22 | 0 | 1.38 | 1.54 | 2.95 |
| P50552 | *VASP* | ID_364 | 22 | 0 | 1.23 | 0.75 | 2.69 |
| Q9BTT0 | *ANP32E* | ID_89 | 22 | 0 | 0.73 | 0.76 | 2.66 |

Note. The top 10 proteins accordant with the profile.


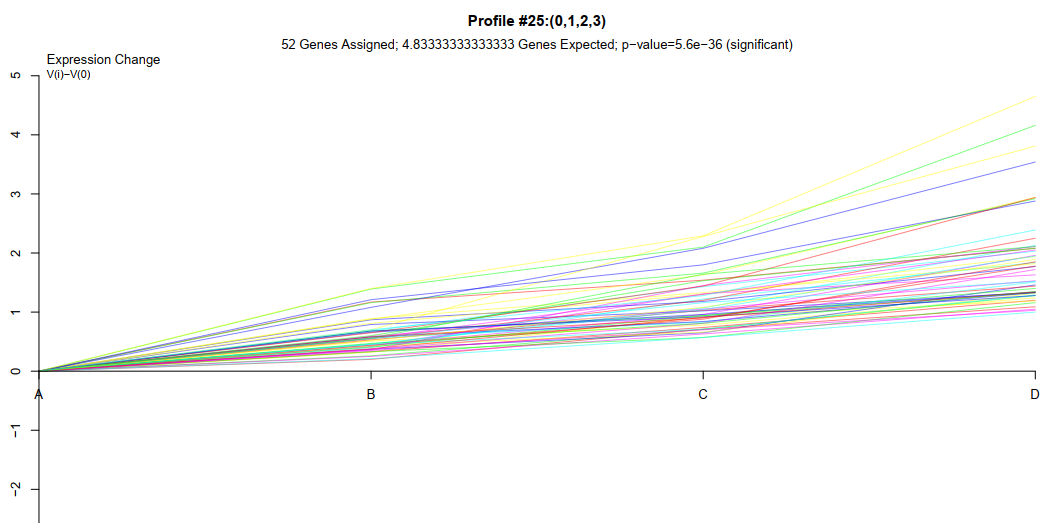


**Supplementary Figure S2. Trend model of protein expressions (Profile #25)**

Note: Each trend model has a graph corresponding to it. In the graph, each line represents a protein which visually reflect the expression changes in the trend model.

**Supplementary Table S2A. Detailed list of proteins in the trend model (Profile #25)**

| **Proteins** | **Gene Name** | **SPOT** | **Profile** | **A** | **B** | **C** | **D** |
| --- | --- | --- | --- | --- | --- | --- | --- |
| P08575 | *PTPRC* | ID_561 | 25 | 0 | 1.4 | 2.29 | 4.65 |
| P25205 | *MCM3* | ID_472 | 25 | 0 | 0.7 | 2.28 | 3.81 |
| Q03518 | *TAP1* | ID_259 | 25 | 0 | 0.88 | 1.63 | 2.94 |
| P09874 | *PARP1* | ID_550 | 25 | 0 | 0.65 | 1.44 | 2.94 |
| P34897 | *SHMT2* | ID_422 | 25 | 0 | 1.16 | 1.66 | 2.92 |
| Q32MZ4 | *LRRFIP1* | ID_178 | 25 | 0 | 0.44 | 0.96 | 1.96 |
| O43390 | *HNRNPR* | ID_641 | 25 | 0 | 0.52 | 1.08 | 1.89 |
| P61225 | *RAP2B* | ID_317 | 25 | 0 | 0.44 | 0.88 | 1.85 |
| Q9H0D6 | *XRN2* | ID_81 | 25 | 0 | 0.79 | 1.02 | 1.33 |
| P49368 | *CCT3* | ID_375 | 25 | 0 | 0.58 | 0.84 | 1.33 |

Note. The top 10 proteins accordant with the profile.

**Supplementary Table S3. Detailed list of 50 proteins selected**

| NO. | Proteins | Gene Name | NO. | Proteins | Gene Name | NO. | Proteins | Gene Name |
| --- | --- | --- | --- | --- | --- | --- | --- | --- |
| 1 | Q96Q06 | *PLIN4* | 18 | Q08945 | *SSRP1* | 35 | P33992 | *MCM5* |
| 2 | P30838 | *ALDH3A1* | 19 | P61978 | *HNRNPK* | 36 | P04792 | *HSPB1* |
| 3 | Q9BXN1 | *ASPN* | 20 | O75369 | *FLNB* | 37 | P0DMV9 | *HSPA1B* |
| 4 | P07305 | *H1F0* | 21 | P41250 | *GARS* | 38 | P51812 | *RPS6KA3* |
| 5 | O15533 | *TAPBP* | 22 | Q13835 | *PKP1* | 39 | P16949 | *STMN1* |
| 6 | P43121 | *MCAM* | 23 | Q9Y6G9 | *DYNC1LI1* | 40 | P08575 | *CD45* |
| 7 | Q9UIJ7 | *AK3* | 24 | P22105 | *TNXB* | 41 | Q13094 | *LCP2* |
| 8 | Q96AC1 | *FERMT2* | 25 | P23229 | *ITGA6* | 42 | P36952 | *SERPINB5* |
| 9 | P55268 | *LAMB2* | 26 | P12109 | *COL6A1* | 43 | P35222 | *CTNNB1* |
| 10 | Q13642 | *FHL1* | 27 | P12111 | *COL6A3* | 44 | Q9HB71 | *CACYBP* |
| 11 | Q9HBL0 | *TNS1* | 28 | P12110 | *COL6A2* | 45 | P27361 | *MAPK3* |
| 12 | Q9NYU2 | *UGGT1* | 29 | P16144 | *ITGB4* | 46 | P29466 | *CASP1* |
| 13 | P62993 | *GRB2* | 30 | P31947 | *SFN* | 47 | P31944 | *CASP14* |
| 14 | Q12874 | *SF3A3* | 31 | P25205 | *MCM3* | 48 | P42224 | *STAT1* |
| 15 | Q07666 | *KHDRBS1* | 32 | P33993 | *MCM7* | 49 | P51692 | *STAT5B* |
| 16 | P05455 | *SSB* | 33 | P49736 | *MCM2* | 50 | Q14566 | *MCM6* |
| 17 | O00159 | *MYO1C* | 34 | P33991 | *MCM4* |  |  |  |

Note. The above 50 were selected according to the results of bioinformatics analysis.
